# Ionized hypercalcemia in 238 cats from a referral hospital population (2009‐2019)

**DOI:** 10.1111/jvim.16627

**Published:** 2023-01-16

**Authors:** Sophie E. Broughton, Dan G. O'Neill, Harriet M. Syme, Rebecca F. Geddes

**Affiliations:** ^1^ Department of Clinical Science and Services, Royal Veterinary College University of London London UK; ^2^ Pathobiology and Population Sciences, The Royal Veterinary College Herts UK

**Keywords:** AKI, calcium, cat, idiopathic, kidney stone

## Abstract

**Background:**

Ionized calcium concentration ([iCa]) is more sensitive for detecting calcium disturbances than serum total calcium concentration but literature on ionized hypercalcemia in cats is limited. Urolithiasis is a possible adverse consequence of hypercalcemia.

**Hypothesis/Objectives:**

To describe clinical details of diagnoses associated with ionized hypercalcemia in cats and association with urolithiasis.

**Animals:**

Cats (238) seen between 2009 and 2019 at a referral hospital with [iCa] above the normal reference interval.

**Methods:**

Observational cross‐sectional study. Signalment, serum biochemical and imaging findings were reviewed for cats with ionized hypercalcemia considered to be clinically relevant (>1.41 mmol/L). Data were summarized by cause of hypercalcemia (i.e., diagnosis).

**Results:**

Diagnoses for the 238 cats with [iCa] >1.41 mmol/L included: acute kidney injury (AKI; 13%), malignancy‐associated (10.1%), idiopathic hypercalcemia (IHC; 10.1%), chronic kidney disease/renal diet‐associated (8.4%), iatrogenic (5.5%), primary hyperparathyroidism (2.1%), vitamin D toxicity (2.1%) and granulomatous disease (1.7%). In 112 cases (47.1%), no cause for ionized hypercalcemia could be determined (n = 95), hypercalcemia was transient (n = 12), or the cat was juvenile (<1 year; n = 5). Urolithiasis was identified in 83.3% of AKI, 72.7% of iatrogenic, 61.1% of CKD/renal diet‐associated and 50% of IHC cases that were imaged (<50% for other diagnoses). Diagnoses with a high proportion of concurrent total hypercalcemia included primary hyperparathyroidism (100%), vitamin D toxicity (100%), malignancy‐associated (71.4%), granulomatous disease (66.7%) and IHC (65.2%).

**Conclusions and Clinical Importance:**

Ionized hypercalcemia was most commonly associated with kidney diseases, neoplasia or IHC. The proportion of urolithiasis cases varied by diagnosis.

**Abbreviations**
AKI: acute kidney injury
CKD: chronic kidney disease
FIP: feline infectious peritonitis
GFR: glomerular filtration rate
IHC: idiopathic hypercalcemia
IRIS: International Renal Interest Society
IQR: interquartile range
[iCa]: ionized calcium concentration
iCa: ionized calcium
PTHrP: parathyroid hormone related peptide
PTH: parathyroid hormone
PUPD: polyuria and polydipsia
RVC: Royal Veterinary College
SUB: subcutaneous ureteral bypass

## 1 INTRODUCTION

Calcium is important for many cellular functions. Although assessment of serum total calcium concentration is available on most routine serum biochemistry panels, ionized calcium (iCa) is the biologically active fraction.^1^ Total hypercalcemia has poor sensitivity for detecting ionized hypercalcemia in cats^2^, ^3^ and therefore [iCa] should be measured directly whenever possible. Ionized hypercalcemia is caused by a number of underlying pathological and physiological processes,^4^ however the relative frequencies of these diagnoses have not been well described. Initial research investigating the causes of hypercalcemia in cats relied on total hypercalcemia.^5^ Subsequently, the condition idiopathic hypercalcemia (IHC) was described,^6^ which is now often cited as the most common cause of ionized hypercalcemia in cats.^4^ A recent study of 119 cats with ionized hypercalcemia did not corroborate this finding, identifying IHC in only 13%.^7^ Twenty‐three percent of that study population had neoplasia, and a substantial proportion (28.6%) with an [iCa] above their normal reference interval had nonpathologic, transient or inconsequential hypercalcemia.^7^ Clinical details of these cats, aside from broad diagnostic groups, are lacking. Therefore, further data from larger groups of cats are required to better understand the current distribution of diseases leading to ionized hypercalcemia.

Previous studies have associated urolithiasis with both total (11% of hypercalcemic cats)^5^ or ionized hypercalcemia (14% of hypercalcemic cats),^8^ but the relationship between hypercalcemia and urolithiasis is complex in both humans and animals.^9^ Unless associated with urinary tract obstruction and acute kidney injury (AKI), urolithiasis is arguably a consequence rather than a cause of hypercalcemia. Calcium oxalate urolithiasis is associated with hypercalciuria,^10^, ^11^ but hypercalcemia is not the sole determinant of hypercalciuria.^9^ Urolithiasis previously has been documented only to occur in 35% of cats with IHC^6^ and only 14% of cats with obstructive ureteral uroliths are reported to have total hypercalcemia,^12^ despite 98% of obstructing ureteroliths being composed of calcium oxalate.^13^ Further information on the causes of ionized hypercalcemia and urolith formation in cats therefore is required.

Our aims were to describe cats with ionized hypercalcemia (cases) in a referral center and to describe the biochemical and signalment differences among causes of ionized hypercalcemia (diagnoses). A secondary aim was to identify the proportion of cases with each diagnosis that had concurrent urolithiasis.

## 2 MATERIALS AND METHODS

An observational cross‐sectional study was performed. Medical records of all cats presented to the small animal referral hospital of the Royal Veterinary College (RVC) between 1st January 2009 and 1st January 2019 were retrospectively searched using the VetCompass online database to identify all cases of ionized hypercalcemia. Ethical approval had been granted by the RVC Ethics Review Committee (SR20181652).

An iterative process was used to develop a set of effective search terms that could be applied within VetCompass™ for capture of unique candidate cats. The full clinical records then were reviewed manually. Final search terms applied were Hypercal*, Hyperparathyroid*, “ionised calcium”, “ionized calcium”, iCa, Ca2+, “Ca++”, PTH, “parathyroid hormone”, “vitamin D”, Pamidronate, and “free calcium”.

During the study period, whole blood iCa measurements were performed immediately after collection according to manufacturer instructions using 3 different point‐of‐care analyzers: NOVA biomedical (CCX Blood Gas s/n Y0240301Z, January 2009‐September 2016), Radiometer ABL800 Flex Analyzer (October 2016‐study end) and iSTAT 1 (Abaxis VetScan 300 V, s/n 705 971, periodic use May 2017‐study end). All iCa measurements were performed using direct ion‐selective electrode potentiometry, but the underlying methodology varied. The reference intervals that informed contemporaneous clinical decision making were 1.13‐1.33 mmol/L (NOVA and Radiometer) and 1.20‐1.32 mmol/L (iSTAT). During data collection, a 95% reference interval was derived for [iCa] (1.26‐1.41 mmol/L) for the Radiometer (Ethical approval was granted by the RVC Welfare and Ethics Committee [URN2019 1919‐2]; see Supplementary Material 1).^14^ The same process previously had been performed on the iSTAT in older cats ([iCa], 1.19‐1.37 mmol/L).^15^ Ionized calcium concentration in humans is not believed to increase with age,^16^ but similar studies in cats are not available. Measurement of [iCa] was performed in some cases at Nationwide Laboratories and Michigan State University Veterinary Diagnostic Laboratory in accordance with their sample requirements. Throughout the study period, the clinical team considered ionized hypercalcemia to be >1.4 mmol/L. Therefore, based on our calculated reference intervals and collective clinical experience, cats with [iCa] ≤1.41 mmol/L were excluded. Cats also were excluded if [iCa] was not recorded or if the cat was documented as hypercalcemic before 1st January 2009.

Signalment data was extracted using standard VetCompass^TM^ methods.^17^ In addition, clinical data was manually extracted to determine visit date when ionized hypercalcemia was reported, [iCa], pH and machine used for measurement of this [iCa], maximum and minimum [iCa] after the first increased [iCa], presenting complaint or reason for referral, medications and diets received before this visit, and results of clinical investigations performed (urinalysis, serum biochemistry, hormonal assessment and imaging). Only data from biochemistry profiles performed on serum samples at the RVC Diagnostic Laboratory were included in analyses (ILAB600, study start to August 2017; Beckman Coulter AU680, August 2017 to study end).

Each case was reviewed by a resident (SEB) and diplomate (RFG) in internal medicine, and assigned a diagnosis largely based on differential diagnoses previously described for ionized hypercalcemia in cats,^4^ using diagnostic criteria presented in Table 1. Diagnoses were retrospectively updated if follow‐up data indicated a different disease than that originally assigned, or if no diagnosis had been recorded in the clinical records. A single diagnosis was assigned per cat.

**TABLE 1:** Definitions of diagnoses for ionized hypercalcemia in cats

Diagnosis	Definition
Acute kidney injury (AKI)	At least 1 iCa >1.41 mmol/L after acute development of azotemia (AKI) or acute worsening of chronic azotemia (acute‐on‐chronic kidney disease) Unless: Previous ionized hypercalcemia had been documented before the occurrence of AKI Hypercalcemia persisted at the same magnitude despite improvement in azotemia Renal neoplasia was identified as the cause of the AKI
Chronic kidney disease (CKD)/renal diet‐associated	At least 1 iCa >1.41 mmol/L after feeding a diet formulated for feline CKD, or hypercalcemia that resolved after cessation of this diet Cats with CKD with iCa >1.41 mmol/L and no other comorbidities identified
Granulomatous	At least 1 iCa >1.41 mmol/L AND Granulomatous inflammation identified on cytology or histopathology OR Granulomas reported on ophthalmic examination
Iatrogenic	At least 1 iCa >1.41 mmol/L that occurred only after administration of oral or intravenous calcium supplementation, or veterinary prescribed vitamin D supplementation Cases with acute kidney injury (AKI) provided iCa was normal or low before administration of calcium
Idiopathic (IHC)	At least 1 iCa >1.41 mmol/L AND No evidence of another disease process after full diagnostic work up Hypercalcemia did not resolve without specific medication to decrease iCa The presence of a normal plasma PTH concentration did not exclude a case from this diagnosis given the variability of assays available if the patient did not develop further clinical signs indicative of another pathological process in the following 3 months
Juvenile	Under 1 year of age iCa >1.41 mmol/L, but ≤1.6 mmol/L No disease process identified that has been associated with ionized hypercalcemia
Malignancy‐associated	At least 1 iCa >1.41 mmol/L AND Diagnosis of a neoplasm associated with hypercalcemia, either by osteolysis (if evident on imaging), primary or metastatic bone neoplasia or parathyroid hormone‐related peptide (PTHrP) (para‐neoplastic) production If a case was treated for presumptive neoplasia without further investigations to confirm PTHrP production or T‐cell lymphoma, it was included if no other cause for the hypercalcemia was identified and hypercalcemia resolved with tumor remission Patients with functional parathyroid neoplasia were not included in this group
Primary hyperparathyroidism	iCa >1.41 mmol/L AND Plasma parathyroid hormone (PTH) concentration above, or in the upper half of the laboratory reference interval Concurrent serum phosphate concentration below or in the bottom half of the reference interval AND/OR Parathyroid nodule on cervical imaging normo‐ or hypo‐calcemic at reassessment postparathyroidectomy Histopathological evidence of a parathyroid adenoma, adenocarcinoma or hyperplasia
Toxicity	At least 1 iCa >1.41 mmol/L AND Increased serum calcitriol or 25‐hydroxyvitamin D concentrations AND/OR With a history of vitamin D exposure Marked hypercalcemia that resolved rapidly with supportive care and where full assessment or necropsy failed to identify another cause
Transient	At least 1 iCa >1.41 mmol/L which resolved without treatment recognized to decrease iCa No disease process identified that has been associated with ionized hypercalcemia
Undetermined	At least 1 iCa >1.41 mmol/L Diagnosis could not be assigned based on the available information OR >1 diagnosis present that is known to cause ionized hypercalcemia and the primary condition could not be determined

Abbreviations: AKI, acute kidney injury; CKD, chronic kidney disease; iCa, ionized calcium; PTH, parathyroid hormone.

International Renal Interest Society (IRIS) stage and the presence or absence of a SC ureteral bypass (SUB™) system additionally was recorded for patients in the chronic kidney disease (CKD)/renal diet‐associated group.^18^ The AKI cases were subcategorized by cause (obstructive versus nonobstructive).

The software SPSS (IBM SPSS Statistics 28.0.0.0), and GraphPad Prism (version 9.4 for macOS, GraphPad Software, San Diego, California; www.graphpad.com) was used for statistical analysis. The 95% confidence intervals were calculated using Epitools.^19^ Numeric data were assessed for normality by inspection of histograms. Descriptive statistics for nonnormally distributed data were expressed as median (interquartile range [IQR]). Chi‐squared analysis was used to compare breed distributions between cases and the overall hospital population. Selected biochemical variables were compared among diagnoses using the Kruskall‐Wallis test with post hoc Dunn's test with Bonferroni correction for multiple comparisons. An adjusted *P* value <.05 was considered significant.

## 3 RESULTS

From a population of 11 431 cats between 1st January 2009 and 1st January 2019, 1448 (12.7%) cats were identified as candidate cases. After manual review, 238/11431 (2.08%) cats met the case definition. Of these, 69.3% (165/238) were nonpurebred, 29.8% (71/238) were purebred cats, and 2 cases (0.8%) were purebred crosses. No difference was detected in the proportion of purebred/purebred crosses in the cohort of cats with ionized hypercalcemia versus the study population (30.67% [73/238] in the hypercalcemia cohort versus 29.3% [3350/11431] in the study population; *P* = .67). The 238 cats with ionized hypercalcemia were allocated to a diagnosis as presented in Table 2, and 34 (14.3%) originally had been referred for investigation of hypercalcemia (total or ionized). The distribution of analyzers used for the [iCa] measurements are presented in Table 3. Serum total calcium, creatinine and phosphate concentrations were measured concurrently with [iCa] in 182 cases, before in 6 cases, and after in 12 cases. In 38 cats, biochemical analysis was not performed at the reference laboratory, and therefore biochemical data were not included in statistical analyses. Multiple [iCa] results were documented in the records of 144/238 (60.5%) cases.

**TABLE 2:** Diagnoses and signalment data for 238 cats with a documented ionized calcium concentration >1.41 mmol/L between 1st January 2009 and 1st January 2019 at a UK referral teaching hospital

					Signalment				
	Number of cases		HCa identified and flagged before referral			Breed		Sex	
Diagnosis	n (%)	95% confidence intervals	n	%	Age in years, median (IQR)	Nonpurebred, n (%)	Purebred/purebred cross, n (%)	Male, n (%)	Female, n (%)
Acute kidney injury	31 (13%)	9.3%‐17.9%	1	3.2%	7.7 (5.4‐11.6)	24 (77.4%)	7 (22.6%)	12 (38.7%; 12 MN)	19 (61.3%; 19 FN)
Chronic kidney disease/renal diet‐associated	20 (8.4%)	5.5%‐12.6%	5	25%	7.2 (4.6‐9.8)	15 (75%)	5 (25%)	7 (35%; 2 ME, 5 MN)	13 (65%; 13 FN)
Granulomatous	4 (1.7%)	0.7%‐4.2%	1	25%	7.5 (2.9‐12.9)	3 (75%)	1 (25%)	3 (75%; 1 ME, 2 MN)	1 (25%; 1 FN)
Iatrogenic	13 (5.5%)	3.2%‐9.1%	1	7.7%	6.5 (4.3‐8.5)	7 (53.8%)	6 (46.2%)	10 (76.9%; 10 MN)	3 (23.1%; 1 FE, 2 FN)
Idiopathic	24 (10.1%)	6.9%‐14.6%	9	37.5%	4.8 (3.6‐10.6)	12 (50%)	12 (50%)	10 (41.7%; 2 ME, 8 MN)	14 (58.3%; 2 FE, 12 FN)
Juvenile	5 (2.1%)	0.9%‐4.8%	0	0%	0.3 (0.3‐0.3)	3 (60%)	2 (40%)	2 (40%; 2 ME)	3 (60%; 3 FE)
Malignancy‐associated	24 (10.1%)	6.9%‐14.6%	6	25%	11.2 (4.8‐13.5)	20 (83.3%)	4 (16.7%)	12 (50%; 1 ME, 11 MN)	12 (50%; 1 FE, 11 FN)
Primary hyperparathyroidism	5 (2.1%)	0.9%‐4.8%	2	40%	15.5 (13.7‐15.6)	4 (80%)	1 (20%)	3 (60%; 3 MN)	2 (40%; 2 FN)
Toxicity	5 (2.1%)	0.9%‐4.8%	4	80%	.6 (.3‐3.2)	2 (40%)	3 (60%)	3 (60%; 2 ME, 1 MN)	2 (40%; 1 FE, 1 FN)
Transient	12 (5%)	2.9%‐8.6%	0	0%	9.6 (3.6‐12.5)	9 (75%)	3 (25%)	6 (50%; 1 ME, 5 MN)	6 (50%; 2 FE, 4 FN)
Undetermined	95 (39.9%)	33.9%‐46.3%	5	5.3%	5.6 (3.5‐10.0)	66 (69.5%)	29 (30.5%)	42 (44.2%; 5 ME, 37 MN)	53 (55.8%; 6 FE, 47 FN)

*Note*: Sex reported as total male (percentage; ME, MN), total female (percentage; FE, FN).

Abbreviations: FE, female entire; FN, female neutered; HCa, hypercalcemia; ME, male entire; MN, male neutered.

**TABLE 3:** Distribution of analyzers used for the measurement of ionized calcium in 238 cats with ionized hypercalcemia (ionized calcium >1.41 mmol/L) between 1st January 2009 and 1st January 2019

Analyzer	First ionized calcium (n)	Maximum ionized calcium (n)	Minimum ionized calcium (n)
Nova	96	18	44
Radiometer	127	34	61
iSTAT	10	7	12
Nationwide Laboratories	1	1	0
Michigan State University Laboratory	0	6	3
Not recorded	4	4	4
Total readings	238	70	124

**FIGURE 1:**
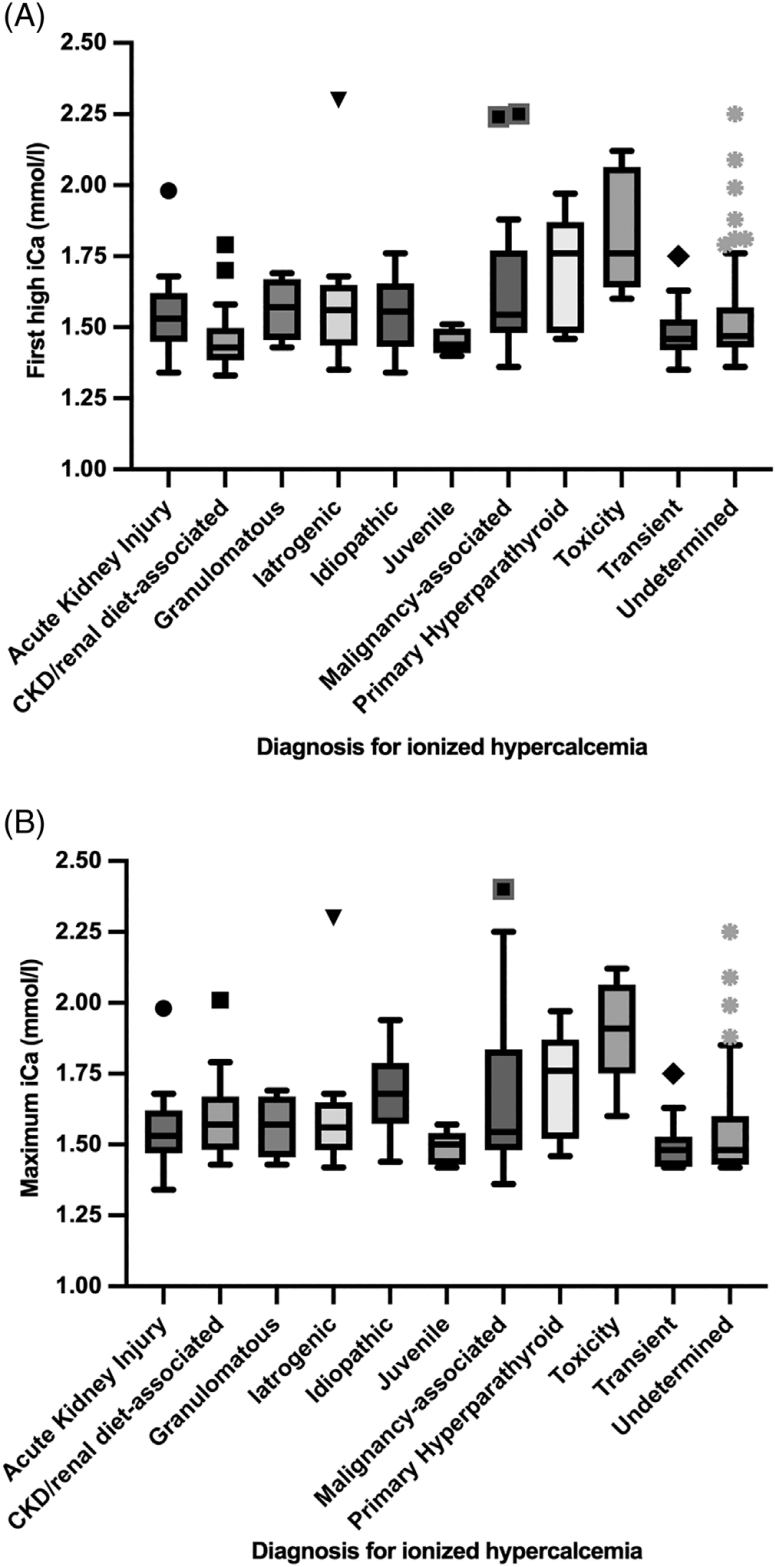
Box and whisker plot (via the Tukey method) of the first high ionized calcium (A) and maximum ionized calcium (B) results from 238 cats with at least 1 ionized calcium >1.41 mmol/L, separated by final diagnosis. The box represents the 25th, median and 75th percentiles, the upper whisker represents the highest data point within 1.5× the interquartile range and the lower whisker represents the lowest data point within 1.5× the interquartile range. Any outliers are plotted individually. CKD, chronic kidney disease; iCa, ionized calcium

A diagnosis was not reached in 95 cats for several reasons: owner declined investigations (n = 16; median [iCa], 1.49 mmol/L; IQR, 1.44‐1.60), clinician decision making (n = 56; median [iCa], 1.46 mmol/L; IQR, 1.43‐1.53) and patient factors (n = 23; median [iCa], 1.53 mmol/L; IQR, 1.43‐1.7) including patient instability precluding diagnostic evaluation, and multiple causes for ionized hypercalcemia identified where it was not possible to determine the primary cause. No difference was found in the [iCa] results among the 3 subgroups (*P* = .17). These cats were designated in Table 2 as “undetermined” and the distribution of [iCa] is represented in Figure 1. They then were removed from further statistical analyses.

Physiological hypercalcemia associated with growth was considered to be the cause of ionized hypercalcemia in 5 cats with a median age 0.29 years (IQR, 0.27‐0.33 years). The maximal [iCa] recorded at any time point for this group was 1.5 mmol/L (IQR, 1.43‐1.54 mmol/L) and only 1 case had concurrent total hypercalcemia.

Clinicopathological data for 138 cats where a diagnosis for their ionized hypercalcemia was reached are presented in Table 4.

**TABLE 4:** Clinical data for 138 cats with clinically significant ionized hypercalcemia presented to a referral center between 1st January 2009 and 1st January 2019 by diagnosis

Variable	Diagnosis									*P* value
	Acute kidney injury	Chronic kidney disease/renal diet‐associated	Granulomatous	Iatrogenic	Idiopathic	Malignancy‐associated	Primary HyperPTH	Toxicity	Transient	
Cases with each diagnosis	n = 31	n = 20	n = 4	n = 13	n = 24	n = 24	n = 5	n = 5	n = 12	
First high iCa (mmol/L)	1.53 (1.45‐1.62)	1.43 (1.39‐1.50)^a,b^	1.57 (1.46‐1.67)	1.56 (1.44‐1.65)	1.56 (1.43‐1.66)	1.55 (1.48‐1.77)^a^	1.76 (1.48‐1.87)	1.76 (1.64‐2.07)^b,c^	1.46 (1.42‐1.53)^c^	<.001
Max iCa (mmol/L)	1.53 (1.47‐1.62)^c,d^	1.57 (1.48‐1.67)	1.57 (1.46‐1.67)	1.56 (1.48‐1.56)	1.68 (1.57‐1.79)^a,c^	1.55 (1.48‐1.84)	1.76 (1.52‐1.87)	1.91 (1.75‐2.07)^b,d^	1.48 (1.42‐1.53)^a,b^	<.001
Biochemistry performed	n = 27	n = 18 (TCa) n = 19 (Crea + PO4)	n = 3	n = 10	n = 23	n = 21	n = 5	n = 5	n = 11	
Serum total calcium (mg/dL)	10.96 (9.96‐12.04)^f,g^	11.26 (10.67‐12.45)	13.6	10.66 (9.03‐12.07)^d,e^	11.72 (10.80‐12.80)	12.68 (11.50‐13.54)^a^	15.32 (13.56‐16.96)^b,d,f^	14.60 (13.64‐16.32)^c,e,g^	9.84 (9.08‐10.24)^a,b,c^	<.001
Total hypercalcemia	11/27 (40.7%)	9/18 (50%)	2/3 (66.7%)	3/10 (30%)	15/23 (65.2%)	15/21 (71.4%)	5/5 (100%)	5/5 (100%)	1/11 (9.1%)	
Serum phosphate (mg/dL)	5.77 (5.77‐7.94)^a^	4.84 (3.81‐6.63)	5.89	8.87 (5.19‐13.49)^b^	4.50 (3.57‐4.74)^a,b^	4.31 (3.80‐6.51)	3.07 (2.70‐5.43)	6.88 (5.63‐7.67)	6.26 (5.05‐7.32)	<.010
Serum creatinine (mg/dL)	3.72 (3.02‐5.91)^b,e,g^	2.57 (1.95‐4.58)^a,d^	1.39	11.75 (2.50‐5.04)^c,f,h^	1.71 (1.54‐2.10)^g,h^	1.45 (1.20‐1.75)^d,e,f^	1.88 (1.29‐2.50)	1.34 (1.14‐2.51)	1.22 (0.76‐1.47)^a,b,c^	<.001

*Note*: Significant differences within rows but between diagnoses are shown with superscript symbols (a‐g), all *P* < .05. Initial iCa, max iCa, serum total calcium, serum phosphate and serum creatinine concentrations reported as median (IQR). Insufficient data was available to calculate an interquartile range for serum total calcium, serum creatinine and serum phosphate concentrations for the granulomatous diagnosis. Reference range for total hypercalcemia was >11.2 mg/dL until August 2017, and >11.6 mg/dL from August 2017 until study end.

Abbreviations: Crea, serum creatinine; HyperPTH, hyperparathyroidism; iCa, ionized calcium; Max iCa, maximum ionized calcium; PO4, serum phosphate; TCa, total calcium.

**FIGURE 2:**
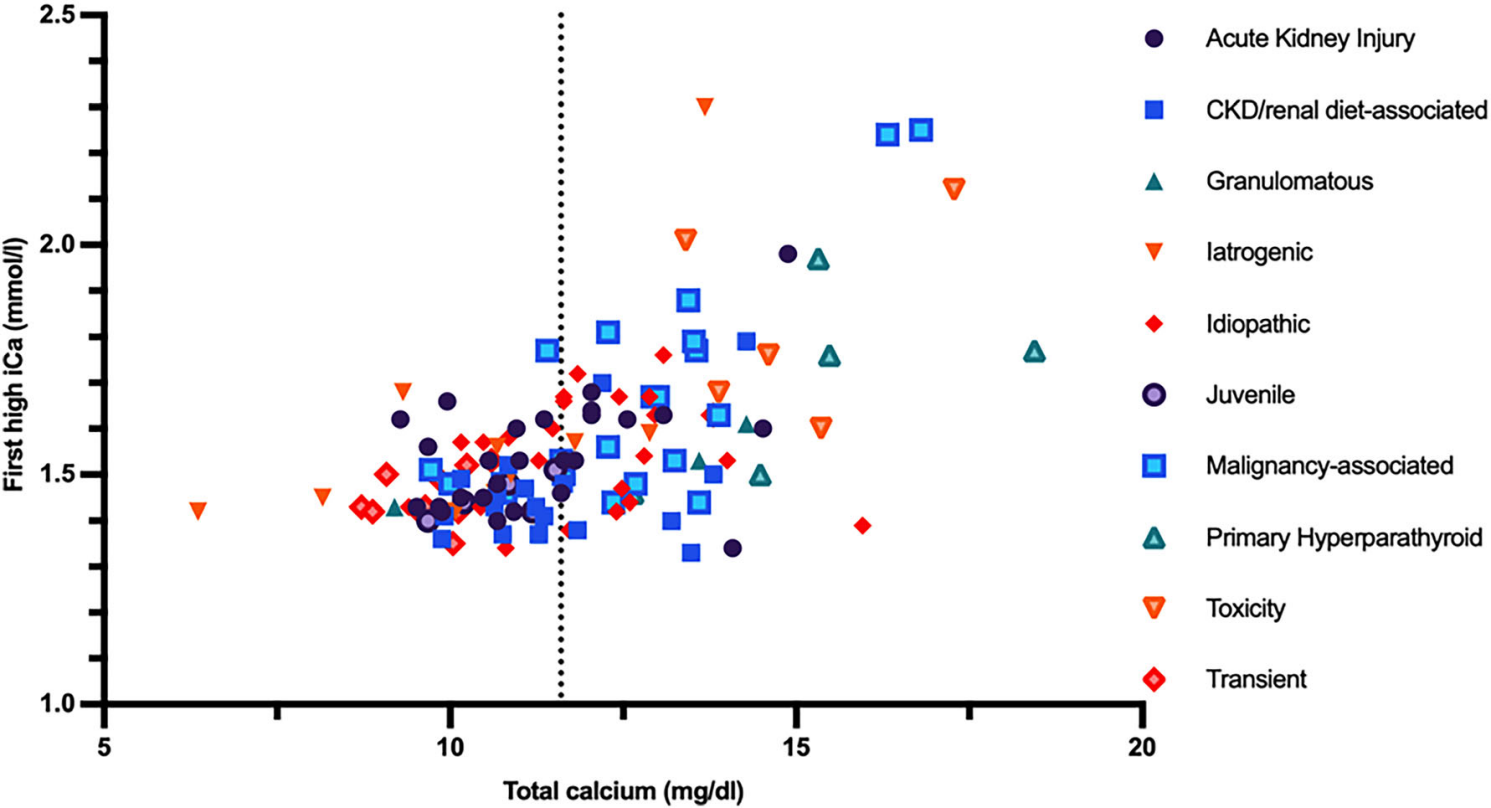
First high ionized calcium concentration and serum total calcium concentration for 128 cats with an ionized calcium >1.41 mmol/L at some point during their investigations, a cause identified for their hypercalcemia, and a serum total calcium concentration measured at the Royal Veterinary College diagnostic laboratory. The dashed line on the X‐axis represents the upper limit of the reference range for serum total calcium concentration (11.6 mg/dL). CKD, chronic kidney disease; iCa, ionized calcium

Twelve (5%) cases had resolution of the hypercalcemia without specific intervention and were designated as having transient hypercalcemia. Only 1/11 had concurrent total hypercalcemia. The median follow‐up for this group was 61 days (IQR, 8.5‐508.5). The magnitude of ionized and total calcium concentrations was lower in this group compared to a number of other etiologies (see Table 4). The distribution of first and maximal [iCa] for each diagnosis is depicted in Figure 1. The correlation between ionized and total calcium concentrations, separated by diagnosis, is depicted in Figure 2.

The most frequent diagnosis for cats with ionized hypercalcemia was AKI (31/238 [13%]). In 27 of 31 cases (87.1%), AKI was associated with urinary tract obstruction, and 15 cats had a SUB™ system (Norfolk Vet Products) placed as part of their management. Of the 27 obstructed cats, 24 had a ureterolith identified and 1 cat had an obstructed SUB™ (previously placed for management of ureterolithiasis). One cat had imaging findings consistent with recent ureteral obstruction that had resolved by presentation, and had previously had a SUB™ placed in the contralateral kidney because of ureterolithiasis. The remaining cat had evidence of a partial ureteral obstruction with no visible ureterolith. Four cats had no evidence of urinary tract obstruction. Two were diagnosed with pyelonephritis, 1 with acute‐on‐chronic CKD with unknown trigger, and 1 developed an AKI after removal of a pancreatic mass.

The next most frequent diagnoses for ionized hypercalcemia were malignancy‐associated and IHC, both in 24/238 (10.1%) of cats. The neoplasms diagnosed were lymphoma or leukemia (n = 11; gastrointestinal, n = 3; central nervous system, n = 3; multicentric, n = 2; leukemia, n = 2; renal, n = 1), epithelial neoplasia (n = 10), plasma cell neoplasia (n = 1), pituitary mass without a histopathological diagnosis (n = 1) and a renal mass that could not be distinguished between lymphoma and carcinoma using immunocytochemistry (n = 1). Three cats with malignancy‐associated ionized hypercalcemia had parathyroid hormone‐related peptide (PTHrP) measured, of which 1 result (4.2 pmol/L) was above the laboratory reference range. This cat was diagnosed with a pulmonary carcinoma.

Of the 24 cats diagnosed with IHC, 21 had PTH measured while under the care of the referral hospital. Several laboratories using different assays and reference ranges were used and therefore these data have not been included for analysis. Sixteen cats had PTH concentrations below the reference interval, and 5 had PTH concentrations within the reference interval, although 3 of these were only just above the lower end of the reference range. The same 21 cats had PTHrP measured, and it was normal in all cases. Fifteen of 23 (65.2%) cases had concurrent total hypercalcemia. The most common reasons for referral for these cats included laboratory abnormalities (11/24), hypercalcemia (9/24), inappetence or anorexia (9/24), abnormalities on imaging (7/24), lower urinary tract signs (6/24), weight loss (6/24) and vomiting (5/24). Only 3/24 (12.5%) cats were presented with polyuria and polydipsia.

Of the 20 cats diagnosed with CKD/renal diet‐associated hypercalcemia, 8 cats (40%) had a SUB™ system in place. One cat without evidence of CKD had hypercalcemia while eating a renal diet, which resolved with diet change. Of the remaining cats, 1 was IRIS stage 1, 10 were IRIS stage 2, 6 were IRIS stage 3 and 2 were IRIS stage 4 when they first had a recorded [iCa] >1.41 mmol/L. Only 50% of the cats in this group had concurrent total hypercalcemia.

Thirteen cats had iatrogenic hypercalcemia, predominantly after calcium gluconate administration for hyperkalemia (usually in association with AKI), although for 1 cat vitamin D over supplementation for hypoparathyroidism was the cause. This group had the highest median serum creatinine concentration, but it was not higher than for the AKI group (iatrogenic hypercalcemia median serum creatinine concentration, 11.76 mg/dL versus AKI, 3.72 mg/dL; *P* = 1.0). Total serum calcium concentration was increased in only 3/10 cats.

Ionized hypercalcemia was attributed to toxicity in 5 cats, 3 of which were diagnosed with hypervitaminosis D with a high suspicion for a single commercial dietary source of intoxication (all presenting between January and June 2010). One cat had a different source of dietary intoxication with vitamin D. In the remaining cat, the toxin was not identified. Five cats were diagnosed with primary hyperparathyroidism. These 2 groups had the most severe ionized hypercalcemia of all diagnoses (both with median [iCa] of 1.76 mmol/L; see Table 4). All cats had concurrent total hypercalcemia. Serum phosphate concentrations varied, but the difference did not reach significance when adjustment for multiple comparisons was made (serum phosphate concentration for toxicity cases, 2.22 mmol/L versus 0.99 mmol/L for primary hyperparathyroidism; *P* = .44). The 2 groups were markedly different in age (0.6 years for toxicity cases versus 15.5 years for primary hyperparathyroidism cases; *P* < .001).

Four cats had granulomatous disease, with no statistical difference detected in biochemical variables from all other groups (see Table 4). Two had a presumptive diagnosis of feline infectious peritonitis (FIP; 1 wet and 1 dry form), 1 had suspected mycobacteriosis and the remaining cat had granulomatous laryngitis with no cause identified.

**FIGURE 3:**
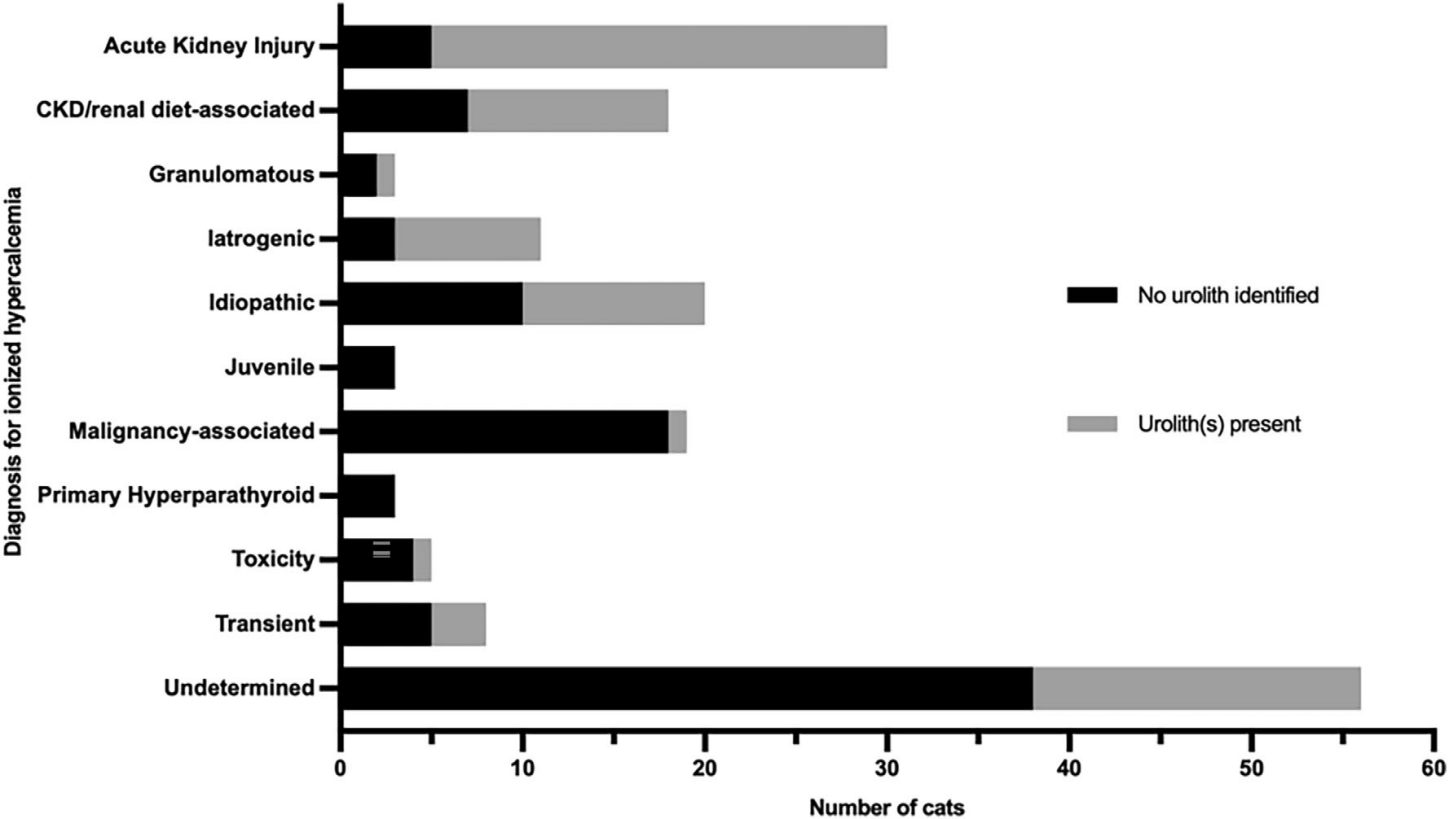
Urolithiasis identified on abdominal imaging in 176 cats with clinically relevant ionized hypercalcemia, by diagnosis. The length of the bar indicates the number of cats with each diagnosis undergoing abdominal imaging, separated by the findings in relation to urolithiasis. CKD, chronic kidney disease

Abdominal imaging was performed in 176 cats when ionized hypercalcemia was first identified. Of these, 78 cats (44.3%) had at least 1 urolith identified. The final diagnoses for the ionized hypercalcemia for cases with and without uroliths on abdominal imaging are depicted in Figure 3. The diagnoses with the most uroliths identified were AKI (83.3%), iatrogenic (72.7%), CKD/renal diet‐associated (61.1%) and IHC (50%). None of the imaged primary hyperparathyroid cases or juvenile cats had uroliths. Of the 78 patients with uroliths identified, 47 had nephrolithiasis, 54 had ureterolithiasis, 25 had cystolithiasis and 5 had urethrolithiasis. All urethroliths and 50 (92.6%) ureteroliths were related to the reason for referral. Only 76% (19) of cystoliths and 63.8% (30) of nephroliths were related to the reason for presentation (ie, many were incidental findings on referral imaging).

## 4 DISCUSSION

We describe the signalment and etiologies for 238 cats presented to a referral center over a 10‐year period with ionized hypercalcemia. Of the 238 cases, 5% had resolution of hypercalcemia without specific intervention and nearly 40% of cases did not have a cause determined. Only 126/238 cats with ionized hypercalcemia had a pathological diagnosis made (either prospectively or retrospectively). The presence of ≥1 uroliths was identified on imaging in 44.3% of 176 cats with ionized hypercalcemia and the uroliths were related to the reason for referral in 70.5%.

Proportions of pathological diagnoses made for ionized hypercalcemia in the cats reported in our study varied slightly from those previously described at Cornell University Hospital for Animals.^7^ However, different hospital‐specific reference ranges were used, and the inclusion and exclusion criteria differed for diagnostic groups. The most frequent diagnosis made in cats with clinically relevant ionized hypercalcemia in our study was AKI (13%), and the majority of cats in this group were urolith‐forming cats with ureteral obstruction. Kidney injury was the second most common cause for ionized hypercalcemia in the previous study of cats at Cornell University Hospital for Animals, but that study combined AKI and CKD cases into a single group with only 5/16 cats with ionized hypercalcemia having AKI.^7^ Acute kidney injury as a cause of hypercalcemia therefore appears to be much higher among the cats investigated in our study. The etiology of AKI in the majority of the cats in our study was ureterolithiasis, and it is possible that the south‐east United Kingdom has a higher incidence of this condition, or that the RVC attracts a higher number of referrals after a diagnosis of this condition in primary care practice. Alternatively, given that the severity of ionized hypercalcemia in AKI patients in our study was mild to moderate, it may be that a number of these cases had instead been placed into the inconsequential/transient hypercalcemia group of the study from Cornell, given that the Cornell study had a higher reference interval for [iCa] (up to 1.47 mmol/L) than in our study.^7^ It also should be considered that in some cats with AKI (attributable to ureterolithiasis), ionized hypercalcemia may have had another etiology, despite improvement in [iCa] in parallel with improvement in serum creatinine concentration (and hence their allocation to this group).

Malignancy‐associated hypercalcemia was identified in 10.1% of cats with clinically relevant ionized hypercalcemia in our study, which is lower than previously reported in cats with ionized (22.7%)^7^ or total (29.6%)^5^ hypercalcemia in referral practice. It is possible that some cats with neoplasia did not receive a final diagnosis because their owners may have declined complete investigations when neoplasia was considered very likely, they were misclassified as having IHC if the neoplastic process was occult at the time of investigation or because many neoplastic cases identified in primary care practice may not subsequently be referred.

Vitamin D toxicity was rarely identified (2.1%) as a cause of clinically relevant ionized hypercalcemia in cats over the time period of our study, and no cases of vitamin D toxicity were identified in cats in previous studies.^5^, ^7^, ^8^ Most of our cases of vitamin D toxicity were associated with consumption of a suspected contaminated batch of kitten food (explaining the statistically younger age of these cats) over a 6‐month period, and a single case was associated with a second contaminated kitten food. Both dietary sources previously have been reported in the veterinary literature.^20^, ^21^ Primary hyperparathyroidism was uncommon in cats with ionized hypercalcemia in our study (2.1%) as previously reported (3%‐5.6%).^5^, ^7^, ^8^ Cats with primary hyperparathyroidism had the highest median age at presentation (15.5 years), similar to a prior case series,^22^ suggesting that primary hyperparathyroidism is largely a disease of middle‐aged to older cats, as seen in dogs,^23^, ^24^ albeit occurring at a much lower frequency.

Only 34 (14.3%) of all cats with ionized hypercalcemia in our study were referred for investigation of hypercalcemia (total or ionized), along with other clinical findings, but 80% of cats with toxicity as a cause of their hypercalcemia had hypercalcemia as a presenting complaint.

Concurrent total hypercalcemia was identified for all diagnoses, and a significant difference in the severity of total hypercalcemia was identified between many of these diagnoses, likely explaining the discrepancy with previous studies that focused on total hypercalcemia.^5^ Low proportions of total hypercalcemia were identified in cases with transient ionized hypercalcemia (9.1%), iatrogenic hypercalcemia (30%), AKI (40.7%) and CKD (50%). Nearly 60% of AKI and 50% of CKD cases therefore would not have been included in previous studies.^5^ In contrast, malignancy‐associated, primary hyperparathyroidism and toxicity had high percentages of cases (>70%) with concurrent total hypercalcemia.

Idiopathic hypercalcemia (IHC) was first described in 2000 in cats from a number of university and private veterinary practices, and the signalment and clinical signs in our study are similar to those previously reported.^6^ Only 12.5% of the current IHC cases were presented with polyuria and polydipsia (PU PD). This observation is consistent with previous reports of low frequency of PU PD in hypercalcemic cats,^6^ and in contrast to what is reported in dogs (43%‐62%).^23^, ^24^, ^25^ The proportion of total hypercalcemia in cases of IHC has not previously been reported. We determined that nearly 35% of cats with IHC (where measured) in our study population did not have concurrent total hypercalcemia and therefore IHC cannot be excluded based on the presence of total normocalcemia. A similar proportion of cats was diagnosed with IHC in our study (10.1%) as compared with the study examining ionized hypercalcemia in cats presented to Cornell University Hospital for Animals (13%),^7^ which is in contrast to the general perception regarding the frequency of this condition in cats with hypercalcemia (i.e., that IHC is the most common cause of ionized hypercalcemia in cats).^4^ It is possible that the cats in our study that had an undetermined cause for their ionized hypercalcemia also could have had IHC and therefore IHC may have been more common than is reported here. Alternatively, some cases may have been misclassified as IHC when another cause for ionized hypercalcemia was present. Regardless, we found IHC to be 1 of the 4 most common diagnoses for cats with ionized hypercalcemia.

Ionized hypercalcemia has been determined to develop after introduction of moderate dietary phosphate restriction in 19% of healthy cats.^26^ Furthermore, 13% of cats with normocalcemic azotemic CKD developed ionized hypercalcemia when fed a more markedly phosphate‐restricted diet,^27^ with resolution possible after dietary change to a moderately phosphate‐restricted diet in 80% of CKD cases.^28^ Feeding the same moderately phosphate‐restricted diet does not decrease [iCa] in cats that already had ionized hypercalcemia before CKD diagnosis,^28^ suggesting that the causes of increased [iCa] in CKD cats are multifactorial. In many cases in our study we could not separate CKD and dietary phosphate restriction as the cause of hypercalcemia, and these cats therefore were considered together as a single diagnosis. A single cat in this group was fed a renal diet without having any evidence of kidney disease and developed an ionized hypercalcemia and hypophosphatemia. Resolution of these abnormalities occurred after transitioning the cat to an appropriate life‐stage diet, although serum phosphate concentrations still remained in the lower half of the reference range. This observation is consistent with recent identification of patients with lower serum phosphate concentration before transitioning to a phosphate‐restricted diet as being at higher risk of total hypercalcemia.^29^

The magnitude of ionized hypercalcemia was only statistically different among 4 diagnoses (cats with toxicity or IHC had higher [iCa] than cats with AKI, and cats with transient hypercalcemia had lower [iCa] than cats with IHC or toxic causes of hypercalcemia). The magnitude of ionized hypercalcemia therefore could not be used to distinguish among diagnoses. Serum phosphate concentrations were only significantly different among 3 diagnoses (cats with AKI or iatrogenic hypercalcemia had higher serum phosphate concentration than the IHC group), likely reflecting the marked acute decrease in glomerular filtration rate (GFR) in both the AKI and the majority of cats in the iatrogenic hypercalcemic groups. Serum phosphate concentrations in the primary hyperparathyroid group were the lowest in our study, which is biologically consistent given the expected increase in fractional excretion of phosphate driven by PTH excess. Interestingly, however, this difference did not reach statistical significance after correction for multiple comparisons, likely reflecting a type II statistical error given the small number of cats in the primary hyperparathyroid group.

The reasons for a number of cases not receiving a confirmed diagnosis for hypercalcemia at the time of consultation or retrospectively are manifold, including owner satisfaction with the suspected diagnosis or unwillingness to further investigate incidentally identified ionized hypercalcemia in a patient with other clinically relevant disease. Clinicians may be reluctant to request expensive calcium regulatory hormone testing unless serum calcium concentrations are markedly increased or the patient has clinical signs that cannot be related to another condition. The number of transient cases identified both in our study and in previous studies emphasizes the need to confirm persistent or clinically relevant ionized hypercalcemia before proceeding with other diagnostic testing.^7^ In many cases, clinicians may not request additional testing after a diagnosis of CKD has been made, because of the recognized difficulty in interpreting PTH results in these cats. A number of CKD cases therefore may have had IHC instead, but lack of prior [iCa] measurement makes this supposition impossible to confirm.

The subset of cats with ionized hypercalcemia that form uroliths previously was considered a separate diagnosis in studies of the etiopathogenesis of hypercalcemia in cats,^5^, ^8^ but we believe doing so underestimates the complexity of this group. Uroliths were identified in a large proportion of cats with clinically relevant ionized hypercalcemia (44.3% of cats that underwent abdominal imaging) in our study, the majority of which had a diagnosis established. The highest proportions of cases with uroliths were seen in the AKI and iatrogenic groups. Interestingly, the majority of cats diagnosed with so‐called iatrogenic hypercalcemia had AKI secondary to ureteral obstruction, but had normocalcemia before administration of calcium gluconate for management of hyperkalemia. The so‐called iatrogenic cats therefore were urolith formers despite not having chronic ionized hypercalcemia. This situation also may have occurred in the majority of the AKI group, but there was no way to retrospectively identify if cats given this diagnosis had ionized hypercalcemia before development of AKI, but improvement or resolution of ionized hypercalcemia after treatment for AKI was a diagnostic criterion for this group. Nevertheless, from our data, it appears that many upper urinary tract urolith‐forming cats with clinically relevant consequences (e.g., ureteral obstruction) do not have prior chronic ionized hypercalcemia, despite the majority of these uroliths being calcium oxalate.^13^ Differences in calcium handling in the kidney therefore may be implicated in urolith formation in these cats, but not necessarily overt systemic hypercalcemia, as is also the case for the majority of urolith formers.^12^ Unfortunately, measurements of urinary fractional excretion of calcium were not available, and would be required to further investigate the association between hypercalcemia and urolith formation. The most common pathogenesis in our AKI group was urinary tract obstruction (87.1%), with 88.9% of these having ureteroliths identified. This finding is in contrast to the causes of AKI in cats reported in the veterinary literature, with the most common cause being nephrotoxicosis (41%‐56%).^30^, ^31^ This observation suggests that hypercalcemia as a result of AKI is not simply associated with decreased GFR, but to some intrinsic difference in the patient.

The CKD/renal diet group encompassed a diverse group of cats including those with idiopathic CKD and cats with known prior renal insults such as postrenal obstruction. Ionized hypercalcemia was identified during routine follow‐up after SUB™ placement in 40% of the CKD group. Therefore, even cats that fall into IRIS stage 1 after ureteral obstruction should have ongoing follow‐up of their [iCa], regardless of their [iCa] at the time of initial presentation. Of those cats imaged, urolithiasis was identified in 50% of cats with IHC, which is higher than the 35% previously reported^6^ and may reflect the increased number of cats imaged in our study, increased awareness of IHC as a differential diagnosis or a true increase in the prevalence of urolithiasis. Interestingly, none of the cats with primary hyperparathyroidism were identified to have uroliths, suggesting that persistent hypercalcemia is not the only factor associated with an increased risk of urolith formation, and further research into the pathogenesis of calcium oxalate urolithiasis is needed.

Our study had some limitations associated with its retrospective design. The inherent inconsistency of data recording in a hospital with changing systems over a 10‐year period and the variable approaches among clinicians led to a number of case exclusions and cats where diagnoses could not be assigned retrospectively. Likely some repeat [iCa] measurements were made but the data has been lost from the paper‐based medical records system. Some cases therefore may have been classified as transient, or excluded because of marginal increases in [iCa], and such cases may have had a pathological cause for hypercalcemia (particularly IHC, where a waxing and waning course is possible). The association between AKI and hypercalcemia is complex and, despite strict definitions, some cats may have been allocated to the AKI group when another cause for ionized hypercalcemia also may have been present. It is also possible that breed and neuter status were incorrectly recorded in the hospital computer system. The selection of cases from a referral hospital likely introduced bias, and we may have identified relatively fewer cases of conditions routinely identified and treated in a primary care setting.

We adjusted for the use of 3 different in‐house machines for [iCa] measurement by applying one hospital‐derived reference range to all results. The upper limit of this calculated reference range was similar to a previously derived in‐house reference range for the iSTAT^15^ and the external laboratories utilized during the study period. However, the use of multiple machines remains a limitation of our study. A further limitation is that 2 different biochemistry analyzers were used for total calcium measurement during the study period, but the bias between the machines was minimal and the reference range at the time of sampling was used to define total hypercalcemia, minimizing the impact on our findings.

Another major limitation was the use of the first increased [iCa] from which a diagnosis was made. The first [iCa] may not have been the most clinically relevant for the patient. For this reason, all [iCa] results were considered in making a diagnosis, and statistical testing was performed on the maximal [iCa] measured for each patient, as well as the first presenting [iCa]. Because of the extended duration of follow‐up in some cases, some patients had multiple possible causes for ionized hypercalcemia, especially cases that initially had iatrogenic hypercalcemia that subsequently developed ionized hypercalcemia. It may have been useful to divide these cases and consider them separately for each condition, but the small numbers of cases in which this situation occurred make it unlikely that our findings would have been affected.

Unfortunately, because of the different laboratories and assays used for PTH and vitamin D analogue measurements, it was not possible to perform statistical analyses for these variables. However, in future studies it could be valuable to follow the PTH concentrations of cats diagnosed with IHC using a single assay to better understand this condition.

In conclusion, AKI, CKD, IHC and malignancy‐associated ionized hypercalcemia all were found to be causes of clinically relevant ionized hypercalcemia. In our study, the number of cases of transient hypercalcemia and the discrepancy between machine‐ and hospital‐specific reference intervals for [iCa] highlight the importance of confirming the persistence and clinical relevance of ionized hypercalcemia before embarking on intensive diagnostic testing, ideally using a reference interval derived specifically for the hospital and machine in use. The proportions of total hypercalcemia for the different diagnoses also emphasize the risk of relying purely on total calcium to determine a cat's calcium status. Uroliths commonly were identified during imaging of hypercalcemic cats in our study and many were not obstructive. Therefore, abdominal imaging should be considered in all cats with persistent ionized hypercalcemia.

## CONFLICT OF INTEREST DECLARATION

Authors declare no conflict of interest.

## OFF‐LABEL ANTIMICROBIAL DECLARATION

Authors declare no off‐label use of antimicrobials.

## INSTITUTIONAL ANIMAL CARE AND USE COMMITTEE (IACUC) OR OTHER APPROVAL DECLARATION

Approved by the Royal Veterinary College (RVC) Ethics Review Committee (SR20181652) and the RVC Welfare and Ethics Committee (URN2019 1919‐2).

## HUMAN ETHICS APPROVAL DECLARATION

Authors declare human ethics approval was not needed for this study.

## Supporting information


**Data S1.** Supporting information
