# Supplementary material for: Ionized hypercalcemia in 238 cats from a referral hospital population (2009‐2019)
Source: J Vet Intern Med. 2023 Jan 16;37(1):80–91. doi: 10.1111/jvim.16627 (PMC9889682; doi:10.1111/jvim.16627)
Supplement: Supplementary file 1 — Data S1. Supporting information [file JVIM-37-80-s001.pdf]

Supplementary Material 1

**A study to establish canine and feline 95% reference intervals for the Radiometer ABL800 FLEX machines in the Queen Mother Hospital for Animals**

Background:

The Radiometer machines in the Emergency Room and the Intensive Care Unit of the Queen Mother Hospital for Animals (QMHA) are used multiple times every day to measure blood gases and biochemical parameters in dogs and cats within the hospital, however, canine and feline specific reference intervals on an appropriate number of healthy patients have not been derived.

Aims:

The aim of this study was to derive 95% reference intervals for the Radiometer ABL800 FLEX machines for cats and dogs.

Methods:

Dogs and cats presenting to the QMHA Blood Donation Service that were deemed to be healthy and suitable for blood donation were eligible for inclusion in the study. Owners of these patients had given signed permission for any blood taken to be used for blood donation, clinical profiles and in research studies. Ethical approval was granted by the RVC Welfare and Ethics Committee (URN2019 1919-2). Residual blood (.3ml) following routine sampling for health checks prior to blood donation was used to run a Radiometer profile. All breeds of cat were eligible for inclusion, but sighthounds were excluded from the canine cohort due to commonly having analytes that fall outside of typical canine reference intervals.  Samples

were collected prospectively from dogs and cats, to enable for calculation of 95% reference intervals, which require a minimum of 39 samples for calculation.[14]

Each variable measured by the Radiometer was assessed separately. Data were inspected visually with histograms to look for potential outliers and assessed for normality using visual inspection of histograms, the Kolmogorov-Smirnov and Shapiro-Wilk tests. Outliers were removed using the Tukey method, then reference intervals were determined using a parametric or non-parametric method as appropriate.[14]

Results:

Radiometer samples were run on 48 dogs and 44 cats between 8/10/2019 and 22/7/2021. Four canine samples were from sighthounds and were not included in analyses. The remaining dog samples included the following breeds: cross breeds (n=12), Labradors (n=10), Staffordshire bull terriers (n=3), German shepherd dogs (n=3), Border collies (n=2), and one each of the following, Airedale terrier, boxer, Doberman, Dogue de Bordeaux, English springer spaniel, field spaniel, flat-coat retriever, German short-haired pointer, golden retriever, Great Dane, Irish setter, Leonberger, miniature schnauzer, Rhodesian ridgeback. There were five entire female dogs, 11 neutered females, 12 entire males and 16 neutered males. The median age was 7 years (range 3-10 years).

The cat samples were from the following breeds: domestic short-haired (n=28), domestic medium-haired (n=2), domestic long-haired (n=5), Maine Coon (n=5), British shorthaired (n=3) and British longhaired (n=1). There were four entire females, 14 neutered females, 5 entire males and 21 neutered males. The median age was 6 years (range 2-10 years).

Parameters were normally distributed. Outliers were removed where required using the Tukey method and 95% reference intervals were calculated (see Appendix 1 for calculation methods).

Reference intervals for each parameter excluding bilirubin are shown in Tables 1 and 2. Values for bilirubin were only present for one canine and six feline samples so this parameter was not included in analyses.

Table 1: 95% reference intervals for canine samples

| Parameter | Unit | n | Lower end 95% RI | Upper end 95% RI | Lower end of previous RI | Upper end of previous RI |
|---|---|---|---|---|---|---|
| **pH** | | 40 | 7.366 | 7.453 | 7.350 | 7.470 |
| **pCO2** | mmHg | 40 | 29.0 | 51.3 | 37.0 | 47.0 |
| **pO2** | mmHg | 43 | 28.9 | 59.9 | 45.0 | 65.0 |
| **Na+** | mmol/l | 42 | 143 | 150 | 140 | 153 |
| **K+** | mmol/l | 42 | 3.6 | 4.4 | 3.6 | 4.6 |
| **Cl-** | mmol/l | 41 | 112 | 122 | 106 | 120 |
| **Ca2+** | mmol/l | 43 | 1.28 | 1.43 | 1.13 | 1.33 |
| **Glucose** | mmol/l | 44 | 4.4 | 6.8 | 4.7 | 7.3 |
| **Lactate** | mmol/l | 41 | .6 | 1.8 | .6 | 2.5 |
| **Creatinine** | µmol/l | 38 | 62 | 105 | 50 | 140 |

Table 2: 95% reference intervals for feline samples

| Parameter | Unit | n | Lower end 95% RI | Upper end 95% RI | Lower end of previous RI | Upper end of previous RI |
|---|---|---|---|---|---|---|
| **pH** | | 43 | 7.302 | 7.437 | 7.350 | 7.470 |
| **pCO2** | mmHg | 43 | 31.5 | 49.4 | 37.0 | 47.0 |
| **pO2** | mmHg | 34 | 32.2 | 58.7 | 45.0 | 65.0 |
| **Na+** | mmol/l | 43 | 150 | 158 | 140 | 153 |
| **K+** | mmol/l | 44 | 3.1 | 4.2 | 3.6 | 4.6 |
| **Cl-** | mmol/l | 43 | 117 | 129 | 106 | 120 |
| **Ca2+** | mmol/l | 42 | 1.26 | 1.41 | 1.13 | 1.33 |
| **Glucose** | mmol/l | 41 | 3.9 | 6.8 | 4.7 | 7.3 |
| **Lactate** | mmol/l | 42 | .3 | 1.8 | .6 | 2.5 |
| **Creatinine** | µmol/l | 37 | 77 | 154 | 50 | 140 |

Appendix 1.

Calculations methods:

Tukey's method for determining outliers:

25th percentile ($Q_1$)

75th percentile ($Q_3$)

IQR calculated

Upper boundary: $Q_3 + 1.5*IQR$

Lower boundary: $Q_1 – 1.5*IQR$

Values outside of these boundaries are consider outliers and should be removed.

Parametric method for 95% reference interval: mean +/- 2*SD

Non-parametric method for 95% reference interval: 2.5th - 97.5th percentile
